# Catarrhal Ozœna

**Published:** 1882-11

**Authors:** 


### CATARRHAL OZŒNA.

Ozœna is probably produced by the action of stagnating secretions, going out from a simple coryxza, on the membranes of the nose. It is therefore advisable to metamorphose by cauterization and the sharp spoon the mucous membrane of the nose.—*Bovel, Revue medicale de la Sussie romane*, 1882. No 5.

